# Multi-Centre UK Analysis of Simultaneous Pancreas and Kidney (SPK) Transplant in Recipients With Type 2 Diabetes Mellitus

**DOI:** 10.3389/ti.2023.11792

**Published:** 2024-02-02

**Authors:** Ruth V. Owen, Harry J. Carr, Claire Counter, Samuel J. Tingle, Emily R. Thompson, Derek M. Manas, James A. Shaw, Colin H. Wilson, Steve A. White

**Affiliations:** ^1^ Institute of Transplantation, Freeman Hospital, Newcastle Upon Tyne, United Kingdom; ^2^ John Radcliffe Hospital, Oxford, United Kingdom; ^3^ Statistics and Clinical Research, NHS Blood and Transplant, Bristol, United Kingdom; ^4^ Blood and Transplant Unit, Newcastle University, Newcastle Upon Tyne, United Kingdom

**Keywords:** equitable access, diabetes mellitus type 2, simultaneous kidney pancreas transplantation, United Kingdom, outcomes

## Abstract

90% of the UK diabetic population are classified as T2DM. This study aims to compare outcomes after SPK transplant between recipients with T1DM or T2DM. Data on all UK SPK transplants from 2003–2019 were obtained from the NHSBT Registry (*n* = 2,236). Current SPK transplant selection criteria for T2DM requires insulin treatment and recipient BMI < 30 kg/m^2^. After exclusions (re-transplants/ambiguous type of diabetes) we had a cohort of *n* = 2,154. Graft (GS) and patient (PS) survival analyses were conducted using Kaplan-Meier plots and Cox-regression models. Complications were compared using chi-squared analyses. 95.6% of SPK transplants were performed in recipients with T1DM (*n* = 2,060). Univariate analysis showed comparable outcomes for pancreas GS at 1 year (*p* = 0.120), 3 years (*p* = 0.237), and 10 years (*p* = 0.196) and kidney GS at 1 year (*p* = 0.438), 3 years (*p* = 0.548), and 10 years (*p* = 0.947). PS was comparable at 1 year (*p* = 0.886) and 3 years (*p* = 0.237) and at 10 years (*p* = 0.161). Multi-variate analysis showed comparable outcomes in pancreas GS (*p* = 0.564, HR 1.221, 95% CI 0.619, 2.406) and PS(*p* = 0.556, HR 1.280, 95% CI 0.563, 2.911). Comparable rates of common complications were demonstrated. This is the largest series outside of the US evaluating outcomes after SPK transplants and shows similar outcomes between T1DM and T2DM recipients. It is hoped dissemination of this data will lead to increased referral rates and assessment of T2DM patients who could benefit from SPK transplantation.

## Graphical Abstract

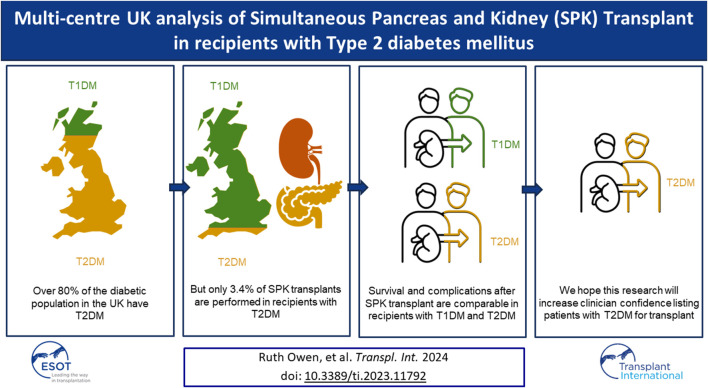

## Introduction

4.9 million people in the United Kingdom (UK) have diabetes characterised by progressive loss of beta-cell mass and/or function. There are broadly two main classifications of diabetes mellitus; Type 1 (T1DM) and Type 2 (T2DM) but sometimes it is difficult to precisely distinguish between the two. The first simultaneous pancreas and kidney transplant was performed in 1966 and initially reserved for patients with T1DM [1]. As the techniques and indications have evolved it was soon realised that some patients with T2DM would also benefit [2–5].

Approximately 90% of the diabetic population have been classified as T2DM compared to only 8% with T1DM [6]. Previously it was thought that T1DM was a disease with onset always in the young, whereas T2DM affected only older adults who were overweight. However, with increasing understanding about diabetes, binary classification of T1DM and T2DM has become increasingly difficult [7]. Studies using historic data comparing various cohorts of diabetic patients is therefore subject to different interpretations when considering the complexities of categorisation. The complex aetiology also makes planning the best management of these patients challenging when they are referred for beta-cell replacement therapy. T2DM is an extremely heterogenous disease. For example, life threatening severe hypoglycaemic unawareness is rare in T2DM but more common in patients with T1DM. Consequently, both Pancreas transplant alone (PTA) and Islet transplant alone (ITA), indicated in the UK solely for recurrent life-threatening hypoglycaemia has never been undertaken for T2DM patients. In the current study outcomes after SPK, as opposed to solitary pancreas transplantation, were investigated in patients with T2DM.

The current UK listing criteria for SPK in a potential T2DM recipient includes; 1) the need for insulin treatment and dependence 2) a BMI of ≤30 kg/m^2^ and 3) patients must be receiving dialysis or have a GFR ≤20 mLs/min [8]. The presence of C-peptide is not an absolute contraindication because of inaccuracies in evaluation in patients with renal failure [9]. In essence potential T2DM recipients need to be fit for surgery, not overtly obese, on insulin treatment with end stage renal disease. Numerous previous studies have shown that patient survival after SPK transplant is superior to those patients on dialysis or those having deceased donor kidney transplant alone (KTA) [10–13].

The aim of this study was to compare outcomes in the NHSBT database between patients with either T2DM or T1DM after SPK transplantation.

## Patients and Methods

NHS Blood and Transplant UK registry data was obtained for all simultaneous pancreas and kidney (SPK) transplants that took place between 2003–2019, *n* = 2,236. Cases where the aetiology of diabetes was missing or had been classified as “other” rather than specifically Type 1 or Type 2 diabetes were excluded, as were recipients who had received a re-transplant, resulting in a final cohort of *n* = 2,154. The type of diabetes was predefined by the centre listing the patient for transplant.

Recipient characteristics; age, sex, body mass index (BMI—categorised by the WHO classification) [14], ethnic group (categorised as white or BAME—black, Asian and minority ethnic), waiting time for transplant, pre-transplant insulin requirements and dialysis status were analysed for variations between our two cohorts. Donor characteristics; age, sex, ethnic group, donor type (DBD/DCD), warm ischaemic time (WIT) and cold ischaemic time (CIT) were also analysed for variation.

Recipient survival and death-censored pancreas and kidney graft survival were analysed at 1, 3, 5 and 10 years. Pancreas graft failure was defined by the recipient follow-up centre based on the resumption of insulin treatment. Kidney graft survival is defined as resumption of dialysis.

We further delineated our groups by BMI into; T1DM < 30 kg/m^2^, T1DM > 30 kg/m^2^, T2DM < 30 kg/m^2^ and T2DM > 30 kg/m^2^ and performed recipient survival and death-censored pancreas and kidney graft and patient survival at 10 years. We also further delineated our groups by ethnic group into; T1DM-White, T1DM-BAME, T2DM-White, T2DM-BAME.

Any patient outside standard listing criteria is discussed through an exemptions panel. Expert opinion within this group guided potential listing.

Common complications after pancreas transplant were analysed between our two cohorts including; incidence of post-operative myocardial infarction (MI), cerebrovascular accident (CVA), anastomotic leak, urinary tract infection (UTI), systemic infection (further delineated into bacterial, viral or fungal), pancreatitis, rejection at 3 months and resumed insulin use at 1 year.

This study aims and methodology were submitted to the NHS Blood and Transplant Research Advisory Group (RAG) and approved prior to gaining access to the registry data.

### Statistical Analysis

Recipient characteristics were delineated by aetiology of diabetes and stratified by age, sex, body mass index (BMI), ethnic group, waiting time on the transplantation list, pre-transplantation insulin requirement, and dialysis status. These are all reported as percentages or means ± standard deviation. Donor characteristics were also delineated by aetiology of diabetes and stratified by age, sex, ethnic group, donor type (DBD/DCD), WIT and CIT and were reported as percentages or means ± standard deviation.

Univariate analysis of pancreas graft, kidney graft and patient survival were performed using Kaplan Meier survival plots and *p*-values derived from the log-rank test. A cox regression model was used for multivariable survival analysis. Our multivariable model was built using variables that had previously been reported to have a detrimental impact on graft or patient survival (cold ischaemic time, dialysis status). The incidence of common post-operative complications underwent chi-squared analysis. All analyses were performed using GraphPad Prism 9.0 and IBM SPSS statistics version 28. All tests were two-sided and *p* values <0.05 were considered significant.

## Results

The majority, (95.6%) of simultaneous pancreas and kidney (SPK) transplants were performed in recipients with Type 1 diabetes mellitus (T1DM) (*n* = 2,060). Only 3.4% (*n* = 94) of SPK transplants have been performed between 2003 and 2019 in recipients with type 2 diabetes mellitus (T2DM). Over the past 15 years we have seen an increasing trend in the percentage of SPK transplants being performed in T2DM recipients (1.6% in 2004 to 5.8% in 2018), Table 1. However, numbers remain comparatively small when compared to T1DM recipients. The median follow-up of all patients in this study was 1900 days, which was until death in 193 patients (8.96%).

**TABLE 1:** Number of SPK transplants performed per year.

Year	T1DM	T2DM
	*n* = 2,060	*n* = 94
2004	62 (98.41%)	1 (1.59%)
2005	86 (97.73%)	2 (2.27%)
2006	114 (94.21%)	7 (5.79%)
2007	174 (97.21%)	5 (2.79%)
2008	136 (96.45%)	5 (3.55%)
2009	132 (95.65%)	6 (4.35%)
2010	133 (99.25%)	1 (0.75%)
2011	140 (95.89%)	6 (4.11%)
2012	150 (95.54%)	7 (4.46%)
2013	158 (93.49%)	11 (6.51%)
2014	147 (96.08%)	6 (3.92%)
2015	138 (94.52%)	8 (5.48%)
2016	126 (91.97%)	11 (8.03%)
2017	132 (95.65%)	6 (4.35%)
2018	131 (94.24%)	8 (5.76%)
2019	101 (96.19%)	4 (3.81%)

T1DM, type 1 diabetes mellitus; T2DM, type 2 diabetes mellitus.

### Clinical Characteristics of Recipients

Recipients with T1DM and T2DM were comparable in terms of time on the waiting list and pre-transplant insulin requirements. Recipients with T2DM were more likely to be older (*p* < 0.0001***), male (*p* < 0.0001***), have a higher BMI (*p* = 0.0223*), be from BAME communities (*p* < 0.0001***), Table 2. Our dataset contained 176 recipients with a BMI >30 kg/m^2^. 168 (95.1%) had T1DM and 8 (4.9%) had T2DM. Those patients outside standard criteria are evaluated within an exemptions committee.

**TABLE 2:** Recipient characteristics.

Recipient characteristic	T1DM	T2DM	*p*-value
Age (years)	41.88 ± 8.33	47.46 ± 787	<0.0001****
Sex (%)			<0.0001****
Male	1,189 (57.7%)	75 (79.8%)	
Female	871 (42.3%)	19 (20.2%)	
BMI (Range)	24.77 ± 3.85 (10–36.9)	25.84 ± 3.76 (19.7–34.4)	0.0223*
Ethnic Group (%)			<0.0001****
White	1,863 (91.1%)	41 (43.1%)	
BAME	182 (8.9%)	54 (56.8%)	
Waiting time for transplant (days)	424.8 ± 369.2	372.9 ± 332.6	0.144
Pre-transplantation Insulin Requirement (units)	44.84 ± 19.39	44.76 ± 21.33	0.974
Dialysis status (%)			0.052
Haemodialysis	693 (33.9%)	40 (42.5%)	
Peritoneal	515 (25.1%)	15 (15.9%)	
Not on dialysis	838 (41.0%)	39 (41.4%)	

Data shown as number or mean ± SD or percentage. BAME, Black, Asian and minority ethnic.

* *p* ≤ 0.05, **** *p* ≤ 0.0001.

### Clinical Characteristics of Donors

Donors were comparable with no statistically significant parameters found between our two cohorts when analysing for donor sex, age, ethnic group and donor type (DBD/DCD). Warm ischaemic time (WIT) and cold ischaemic time (CIT) were also similar between the two groups, Table 3.

**TABLE 3:** Donor characteristics.

Donor characteristic	T1DM	T2DM	*p*-value
Age (years)	34.88 ± 13.44	36.91 ± 13.86	0.166
Sex (%)			0.198
Male	1,038 (50.4%)	41 (43.6%)	
Female	1,021 (49.6%)	53 (56.4%)	
Ethnic Group (%)			0.842
White	1,892 (93.9%)	84 (93.3%)	
BAME	124 (6.1%)	6 (6.7%)	
Donor Type (%)			0.562
DBD	1,692 (82.1%)	75 (79.8%)	
DCD	368 (17.9%)	19 (20.2%)	
Warm Ischaemic Time (mins)	45.39 ± 71.61	53.15 ± 83.81	0.420
Cold Ischaemic Time (mins)	709.3 ± 252.6	756.3 ± 224.6	0.059

Data shown as number + percentage. BAME, Black, Asian and minority ethnic; DBD, donation after brainstem death; DCD, donation after circulatory death.

### Univariate Analysis of the Impact of Diabetes Aetiology on Graft and Patient Survival

**FIGURE 1:**
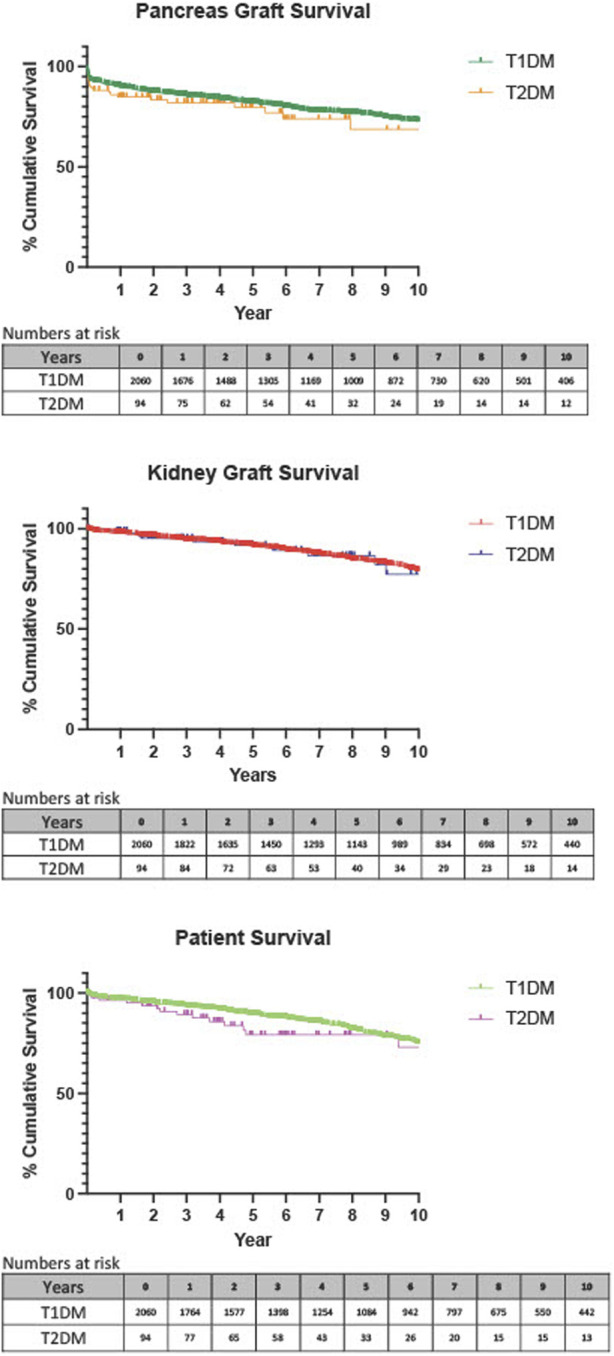
Death-censored Kaplan-Meier analysis of 1 year pancreas graft, kidney graft and patient survival, T1DM recipients compared with T2DM recipients.

Death-censored survival analyses were performed using Kaplan Meier plots and revealed no statistically significant difference in pancreas graft survival at 1 year (*p* = 0.120), 3 years (*p* = 0.316), 5 years (*p* = 0.451), or 10 years (*p* = 0.196), Figure 1. There were also comparable rates of kidney graft survival at 1 year (*p* = 0.438), 3 years (*p* = 0.548), 5 years (*p* = 0.920), and 10 years (*p* = 0.947), Figure 1. When analysing patient survival, again no statistically significant difference was seen at 1 year (*p* = 0.886), or 3 years (*p* = 0.237), Figure 1. There was a statistically significant difference at 5 years (*p* = 0.028*), which showed poorer survival in those with T2DM. This trend was not borne out long-term as survival rates were comparable at 10 years (*p* = 0.161). *p* values and percentage survival were amalgamated into Table 4.

**TABLE 4:** Percentage graft and patient survival at 1, 3, 5, and 10 years.

		T1DM	T2DM	*p*-Value
Pancreas Graft Loss	1 year	89.9%	84.9%	0.120
		(95% CI 91.1–88.5)	(95% CI 90.7–75.8)	
		*n* = 1,676	*n* = 75	
	3 years	85.4%	82.0%	0.316
*n* = number at risk		(95% CI 86.9–83.7)	(95% CI 88.6–72.2)	
		*n* = 1,305	*n* = 54	
	5 years	81.8%	79.8%	0.451
		(95% CI 83.5–79.9)	(95% CI 87.2–69.0)	
		*n* = 1,009	*n* = 32	
	10 years	72.7%	68.7%	0.196
		(95% CI 75.1–70.1)	(95% CI 80.8–51.8)	
		*n* = 406	*n* = 12	
Kidney Graft Survival	1 year	97.6%	98.9%	0.438
		(95% CI 98.2–96.8)	(95% CI 99.8–92.3)	
		*n* = 1822	*n* = 84	
	3 years	92.2%	95.2%	0.548
		(95% CI 93.2–91.0)	(95% CI 98.2–87.7)	
		*n* = 1,450	*n* = 63	
*n* = number at risk	5 years	91.3%	91.7%	0.920
		(95% CI 92.6–89.8)	(95% CI 96.3–82.1)	
		*n* = 1,143	*n* = 40	
	10 years	78.9%	77.2%	0.947
		(95% CI 81.3–76.2)	(95% CI 88.3–58.3)	
		*n* = 440	*n* = 14	
Patient Survival	1 year	96.8%	96.5	0.886
		(95% CI 97.5–95.5)	(95% CI 98.8–89.6)	
		*n* = 1764	*n* = 77	
	3 years	93.2%	89.3%	0.237
*n* = number at risk		(95% CI 94.3–91.9)	(95% CI 94.5–79.6)	
		*n* = 1,398	*n* = 58	
	5 years	89.4%	79.2%	0.028*
		(95% CI 90.8–87.8)	(95% CI 87.6–66.2)	
		*n* = 1,084	*n* = 33	
	10 years	74.8%	73.1%	0.161
		(95% CI 77.4–71.9)	(95% CI 85.0–54.7)	
		*n* = 442	*n* = 13	

Data shown as percentage with 95% confidence intervals. Number at risk depicts how many recipients with follow up at that time period.

* *p* ≤ 0.05.

**FIGURE 2:**
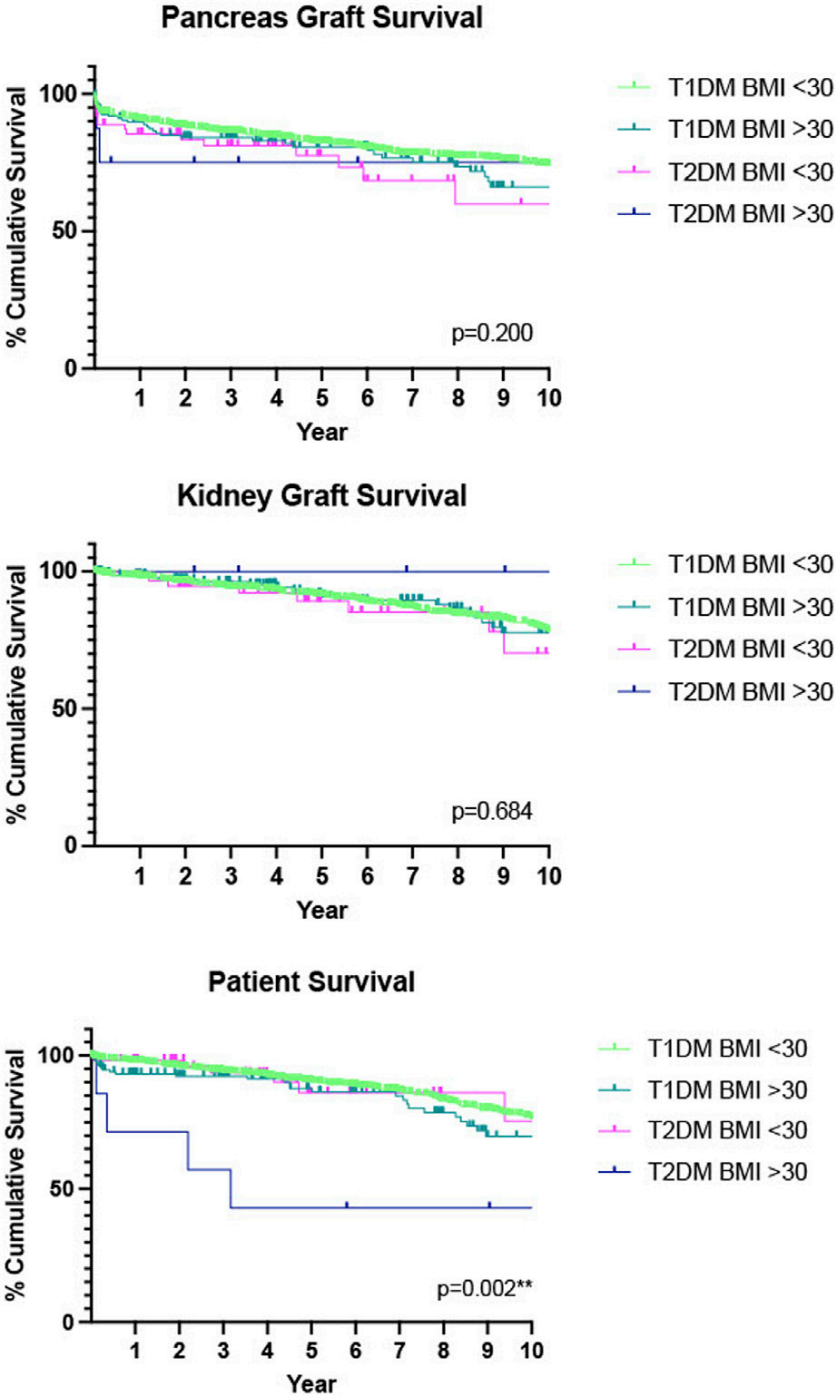
Death-censored Kaplan-Meier analysis of 10 years pancreas graft, kidney graft and patient survival, T1DM recipients compared with T2DM recipients, further stratified by BMI. *n* = 528 had missing data for BMI so were not included in this analysis.

A further analysis was performed further stratifying the two diabetes groups into those with a BMI ≤30 kg/m^2^, and those with a BMI >30 kg/m^2^. A complete case analysis was used and cases without information pertaining to BMI were excluded. In total 176 (8.2%) recipients had a BMI >30 kg/m^2^. Of the 176, 168 (95.5%) had T1DM and 8 (4.5%) had T2DM, Table 5. Although numbers are small in T2DM patients there was no statistically significant difference in pancreas graft (*p* = 0.200) or kidney graft (*p* = 0.684) survival was found between these groups. However, a statistically significant decrease in patient survival was seen in our recipients with T2DM and a BMI >30 compared with the other categories. (*p* = 0.002**), Figure 2.

**TABLE 5:** WHO classification of recipient BMI.

Category	BMI (kg/m^2^)	T1DM	T2DM
		*n* = 1,614	*n* = 72
Underweight	16–18.5	39 (2.42%)	1 (1.39%)
Normal	18.5–25	853 (52.85%)	29 (40.28%)
Overweight	25–30	554 (34.32%)	34 (47.22%)
Obese	>30	168 (10.41%)	8 (11.11%)

Data shown as mean ± SD or percentage.

**FIGURE 3:**
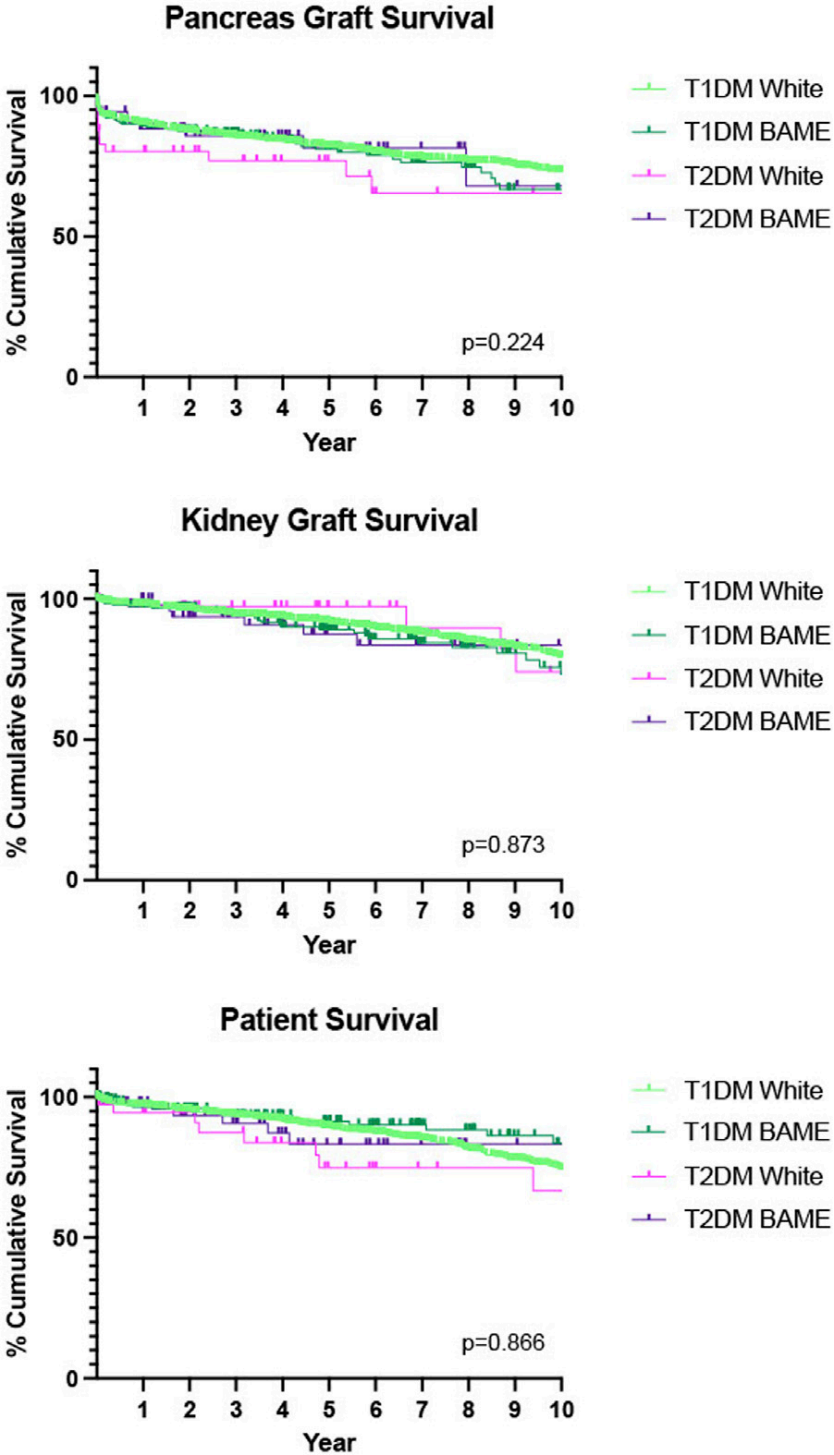
Death-censored Kaplan-Meier analysis of 10 years pancreas graft, kidney graft and patient survival, T1DM recipients compared with T2DM recipients, further stratified by ethnicity. W-White, BAME–Black, Asian and minority ethnic.

We also delineated our diabetes groups by ethnicity and found comparable outcomes for pancreas graft survival (*p* = 0.224), kidney graft survival (*p* = 0.873) and patient (*p* = 0.866) survival, Figure 3.

### Multivariate Analysis of the Impact of Diabetes Aetiology on Graft and Patient Survival

It is important to understand the impact of the type of diabetes within the context of multiple donor and recipient factors. As such, a multivariate analysis was built, including parameters known to influence recipient outcomes. Diabetes type in this multivariate analysis showed no statistically significant impact on pancreas graft survival (HR 1.221, 95% CI 0.619–2.406, *p* = 0.564) Table 6, kidney graft survival (HR 0.953, 95% CI 0.372–2.439, *p* = 0.920), Table 7, or patient survival (HR 1.280, 95% CI 0.565–2.911, *p* = 0.556) Table 8. The multivariate did show that recipient age (HR 0.965, 95% CI 0.951–0.980, *p* < 0.001), recipient BMI (HR 1.049, 95% CI 1.016–1.082, *p* = 0.004) and donor age (HR 1.008, 95% CI 1.008–1.029, *p* < 0.001) were statistically significant variables that affected pancreas graft survival. Recipient age also statistically significantly affected kidney graft survival (HR 0.973, 95% CI 0.955–0.991, *p* = 0.003) and patient survival (HR 1.042, 95% CI 1.024–1.061, *p* < 0.001).

**TABLE 6:** Multivariable analysis of pancreas graft survival.

Variable	Hazards ratio	95.0% CI	*p*-value
Recipient Age	0.965	(0.951–0.980)	<0.001***
Recipient BMI	1.049	(1.016–1.082)	0.004**
Recipient Sex	0.850	(0.665–1.087)	0.195
Recipient Ethnicity	0.808	(0.533–1.226)	0.316
Dialysis Status	1.162	(0.812–1.073)	0.284
Type of Diabetes	1.221	(0.619–2.406)	0.564
Donor Age	1.019	(1.008–1.029)	<0.001***
Donor BMI	0.995	(0.960–1.032)	0.787
Donor Sex	0.935	(0.729–1.200)	0.598
Donor Ethnicity	0.928	(0.580–1.485)	0.757
Donor Type (DCD Vs. DBD)	0.917	(0.660–1.274)	0.605
Warm Ischaemic Time (mins)	1.000	(0.999–1.001)	0.844
Cold Ischaemic Time (mins)	1.001	(1.000–1.002)	0.010

BMI, body mass index; DBD, donation after brainstem death; DCD, donation after circulatory death.

** *p* ≤ 0.01, *** *p* ≤ 0.001.

**TABLE 7:** Multivariable analysis of kidney graft survival.

Variable	Hazards ratio	95.0% CI	*p*-value
Recipient Age	0.973	(0.955–0.991)	0.003
Recipient BMI	1.015	(0.974–1.058)	0.472
Recipient Sex	0.729	(0.545–0.975)	0.033
Recipient Ethnicity	0.939	(0.568–1.552)	0.806
Dialysis Status	1.129	(0.840–1.517)	0.420
Type of Diabetes	0.953	(0.372–2.439)	0.920
Donor Age	1.013	(1.000–1.025)	0.045
Donor BMI	1.000	(0.958–1.044)	0.991
Donor Sex	1.057	(0.780–1.432)	0.722
Donor Ethnicity	0.953	(0.486–1.867)	0.887
Donor Type (DCD Vs. DBD)	1.183	(0.801–1.748)	0.398
Warm Ischaemic Time (mins)	1.001	(1.000–1.002)	0.122
Cold Ischaemic Time (mins)	1.024	(0.976–1.073)	0.336

BMI, body mass index; DBD, donation after brainstem death; DCD, donation after circulatory death.

**TABLE 8:** Multivariable analysis of patient survival.

Variable	Hazards ratio	95.0% CI	*p*-value
Recipient Age	1.042	(1.024–1.061)	<0.001**
Recipient BMI	1.017	(0.981–1.055)	0.349
Recipient Sex	1.229	(0.920–1.641)	0.163
Recipient Ethnicity	0.488	(0.246–0.965)	0.039
Dialysis Status	1.840	(0.692–0.967)	0.788
Type of Diabetes	1.280	(0.563–2.911)	0.556
Donor Age	1.009	(0.997–1.021)	0.164
Donor BMI	0.988	(0.947–1.030)	0.558
Donor Sex	0.900	(0.667–1.213)	0.487
Donor Ethnicity	0.728	(0.372–1.428)	0.356
Donor Type (DCD Vs. DBD)	0.818	(0.523–1.279)	0.379
Warm Ischaemic Time (mins)	1.000	(0.998–1.001)	0.839
Cold Ischaemic Time (mins)	1.001	(1.000–1.001)	0.117

BMI, body mass index; DBD, donation after brainstem death; DCD, donation after circulatory death.

** p ≤ 0.01.

### Incidence of Complications Stratified by Diabetes Aetiology

Complications after transplantation can pose a significant burden on the recipient as well as affect survival outcomes. Common complications after pancreas transplant, including incidence of post-operative myocardial infarction (MI), cerebrovascular accident (CVA), anastomotic leak, urinary tract infection (UTI), systemic infection (further delineated into bacterial, viral or fungal), pancreatitis, rejection at 3 months and resumed insulin use at 1 and 5 years. There was no statistically significant difference between recipients with T1DM or T2DM between any of the above parameters, Table 9. Incidence of graft failure caused by vascular thrombosis was analysed. 184 grafts failed in the T1DM group within 120 days of transplant. Of these 61 (33%) were due to vascular thrombosis. In the T2DM group 11 grafts failed and 2 (18%) were due to vascular thrombosis.

**TABLE 9:** Analysis of common complications after SPK transplants.

Complications three month follow up	T1DM	T2DM	*p*-value
	*n* = 2,060	*n* = 94	
Myocardial Infarction	15	1	0.715
Cerebrovascular Accident	9	0	0.520
Anastomotic Leak	64	2	0.574
UTI	97	9	0.804
Systemic Infection			
- Viral	9	1	0.371
- Bacterial	66	5	0.176
- Fungal	12	0	0.453
Pancreatitis	49	2	0.895
Rejection at 3 months	133	5	0.556
One year follow up	*n* = 1,084	*n* = 33	
Resumed insulin use at 1 year	113	3	0.401
Five year follow up	*n* = 442	*n* = 13	
Resumed insulin use at 5 years	104	1	0.163

UTI, urinary tract infection.

## Discussion

In the UK categorisation of diabetes is primarily a clinical diagnosis. For a diagnosis of T1DM, the current criteria includes; hyperglycaemia (random plasma glucose >11 mmol) with one or more of the following features; ketosis, rapid weight loss, age <50, BMI < 25 kg/m^2^ and/or a personal/family history of autoimmune disease [15]. For a diagnosis of T2DM, the patient should have persistent hyperglycaemia (inferred using a HbA1c > 48 mmol/mol as a surrogate marker) [16], symptoms of; polyuria, polydipsia, unexplained weight loss, recurrent infections or tiredness in the context of known risk factors (i.e., obesity, family history, ethnicity, metabolic syndrome) and the absence of T1DM features (i.e., rapid onset, young age, insulin dependence, ketoacidosis). Unlike other countries, biomarkers such as auto-antibodies or c-peptide are not routinely used for diagnosis or classification in the United Kingdom. The National Institute of Health and Care Excellence (NICE) guidance on diagnosis of diabetes (updated in 2022) recommends using clinical features to make the diagnosis of diabetes, to not routinely use C-peptide, and if using diabetes-specific autoantibodies to take into account the false negative rate of this test [15].

Irrespective of how a patient’s diabetes is classified the unifying result is hyperglycaemia which leads to down-stream micro and macrovascular complications. Early detection and changing medical management of diabetes mellitus undoubtedly helps delay the onset of complications associated with hyperglycaemia however, retinopathy, vasculopathy and nephropathy still remain serious and common afflictions in these patients [6]. Pancreas transplant remains the only realistic, long-term insulin-independent treatment for diabetes [10]. During 2020 there were 198 patients on the UK SPK transplant waiting list compared to 16 who need simultaneous islet and kidney (SIK) transplant [17]. Although the indications are the same the patient groups are likely to be different in terms of associated co-morbidities. SPK transplant is the favoured treatment in those that are fit when considering long-term insulin independence, this also applies to those patients with T2DM.

This study has shown comparable death censored pancreas graft survival and kidney graft survival after simultaneous pancreas and kidney transplants regardless of type of diabetes with the caveat that the diagnosis and type of diabetes was pre-defined by the listing centre using the UK criteria highlighted above. This study has also shown comparable patient survival at 1 and 3 years regardless of diabetes type. Interestingly at 5 years we see a statistically significant decrease in T2DM patient survival when compared to their T1DM counterparts. This trend is not borne out at 10 years which again shows no statistically significant difference in patient survival. We believe this may be partly explained by the older, heavier T2DM having a poorer initial patient survival and the younger, lighter T2DM recipients surviving out to 10 years.

In 2020, an American single-centre study (*n* = 323) demonstrated comparable outcomes in terms of pancreas graft survival (death censored) and incidence of post transplantation diabetes mellitus (PTDM) between recipients with T2DM (*n* = 39) compared with T1DM (*n* = 284). Patients in this study were defined as T1DM and T2DM using clinical assessment at the time of initial evaluation as well as utilising a novel scoring system assessing; pre-transplant insulin requirement, pre-transplant fasting c-peptide levels (assigning a score of +2 if C-peptide <0.5 ng/L, −1 if 0.5–2 ng/L and −2 if >2 ng/L), family history and the presence of diabetes-associated antibodies. A score from −9 to +9 was created, and a negative score correlated with T2DM and a positive score with T1DM [18]. This scoring system was not used in our study.

The largest reported study to date (*n* = 6,756), utilised the United Network for Organ Sharing (UNOS) database. Again, the majority of patients (90.8%, *n* = 6,141) had T1DM and only 8.2% of SPK transplant’s were performed in T2DM (*n* = 582). This study also showed no statistically significant difference in graft and patient survival in patients with T2DM [19]. Type of diabetes was predefined by the listing centre, and no further information regarding this was offered in this publication which makes it harder to draw any more useful detail between the two cohorts.

A smaller single centre study was reported in 2013 by an Austrian group (*n* = 248) comparing T1DM undergoing SPK transplant (*n* = 195) with T2DM SPK transplant (*n* = 21) and T2DM kidney transplant alone (KTA) (*n* = 32) [20]. They defined T2DM using detectable C-peptide levels. This study demonstrated comparable rates of graft survival between T1DM and T2DM recipients undergoing SPK. A statistically significant difference in patient survival was seen when comparing T2DM recipients (both SPK and KTA) with T1DM who underwent SPK (*p* < 0.001). This finding contrasts with the other literature discussed. It is also important to note this paper does not differentiate KTA by donor brain death (DBD), donor circulatory death (DCD) or living related donor (LRD) making it difficult to interpret. However, there is a large American study utilising a National Registry for T2DM patients (*n* = 37,117) where T2DM recipients were shown to have better statistically significant patient survival and kidney allograft survival after SPK when compared to those receiving a KTA alone, irrespective of whether the kidney was from a deceased donor or living donor [21].

A further single centre study in Argentina (*n* = 45), showed no statistically significant difference in patient survival when comparing T1DM (*n* = 35) to T2DM (*n* = 11) after SPK. They classified patients type of diabetes clinically; those who were diagnosed in childhood, with a lower BMI and requiring immediate insulin treatment were classified as T1DM whereas patients who were diagnosed with diabetes aged >30 years/old and with metabolic features were classified as T2DM.

A final study from Washington classifying diabetes by C-peptide >/<0.8 ng/mL (*n* = 136) showed comparable outcomes between their Type and Type 2 recipients. They state that C-peptide status does not influence outcomes after SPK transplant and this treatment option should be offered regardless of their C-peptide level [22].

Whilst the majority (90%) of the UK diabetic population have T2DM, only 3.4% of this population had an SPK. Other countries have comparable proportions of T2DM; in the US 91% of the diabetic population have T2DM [23], in Germany 90%–95% [24] and 90% in the Netherlands [25]. In 2010 the International Pancreas Transplant Registry, IPTR (which receives data from both UNOS and Eurotransplant) showed 8% of SPK’s were performed in patients presumed to have T2DM [26, 27]. Despite comparable proportions of T2DM within these national populations one can extrapolate that the percentage of SPKs performed in recipients with T2DM in the UK is well below that of our American and other European counterparts [3, 28]. However we accept there is no uniform consensus on the criteria used for a diagnosis of T2DM which could explain this observation.

From 2019–2021 a consensus group was formed to deliberate on current pancreas transplant outcomes in an effort to provide evidence to support current practice (28), After removal of duplicate papers and by applying exclusion criteria, 31 studies regarding SPK in T2DM patients were reviewed. The consensus concluded that SPK transplant improved both quality of life and long-term survival in suitable T1DM and T2DM recipients. For T2DM, the authors state that evidence is insufficient to suggest SPK transplant provides greater survival when compared with living donor kidney transplant alone. We did not analyse solitary kidney transplants in this study and to our knowledge this analysis has never been done. It would be interesting to see if SPK transplant is better than PAK transplant with a living donor kidney for patients with T2DM. Numbers in our national dataset would be too small for a useful comparison.

From our cohort we can see that those patients who received a pancreas transplant with T2DM were more likely to be older, male and with a higher BMI. Factors associated with the development of T2DM are obesity and smoking [29–31] which have been typically associated with a male cohort [32, 33]. Currently the UK SPK transplant patient selection policy contains a selection criteria of a BMI <30 kg/m^2^ for T2DM recipients and does not define a BMI restriction for those with T1DM [8]. Although there is some selectively amongst UK centres where >30 kg/m^2^ may be considered as a relative contra-indication by some.

In total 8.2% (*n* = 176) of our cohort had a BMI > 30 kg/m^2^. A previous study from our group showed that whilst BMI does affect outcomes, those who received a pancreas transplant (SPK, PTA and PAK) with a BMI > 30 kg/m^2^ had comparable outcomes with recipients with a BMI < 30 kg/m^2^ and concluded that assigning a cut off of <30 kg/m^2^ as a gatekeeper to pancreas transplantation had the potential to prevent good candidates accessing this treatment option [18,34]. For our study we delineated BMI by type of diabetes to better analyse the data in the context of this selection policy. 8.5% (*n* = 8) of the SPK transplants performed in T2DM had a BMI > 30 kg/m^2^. As this goes against the standard selection policy their case went to an exemptions panel prior to being placed on the waiting list and so were excellent candidates in terms of other parameters. We saw no statistically significant difference in overall graft survival, however recipients with T2DM and a BMI > 30 kg/m^2^ had poorer patient survival than the other categories. Whilst numbers are small in our T2DM > 30 kg/m^2^ group, this would suggest that the combination of T2DM and obesity is of concern. Many T2DM patients are not obese and, in this study, have been shown to have comparable outcomes. Those with T1DM showed comparable graft and patient survival outcomes independent of BMI.

Our study also found that those patients with T2DM receiving a transplant were much more likely to be from a BAME (Black, Asian and minority ethnic) community rather than their T1DM counterparts. This is not unsurprising because from epidemiological studies we know that UK BAME communities have a 3–5× higher prevalence of T2DM with an earlier age of onset [28, 35, 36]. We further analysed our dataset to better understand the role ethnicity played in recipient outcomes. We found no statistically significant difference in either graft or patient survival regardless of ethnicity.

An analysis of common complications after pancreas transplantation was performed. This included incidence of myocardial infarction, cerebrovascular accident, anastomotic leaks, urinary tract infections, systemic infections, pancreatitis, graft rejection at 3 months and resumed insulin use (at 1 year and also 5 years). Both T1DM and T2DM have been associated with an increased incidence of infection [37], poorer wound healing [38] and thrombotic events [39, 40]. When compared against each other we found comparable rates of all complications regardless of type of diabetes.

### Study Limitations

This study is limited by certain factors. Our T2DM cohort was a small highly selected group (3.4%) relative to the UK’s overall population of T2DM patients. However, as we had 96 patients we believe this to be sufficiently high to allow some useful conclusions to be made.

The type of diabetes was predefined by recipient centres using clinical judgement as described above. Unlike other countries objective measurements such as C-peptide levels and presence of antibodies were not routinely utilised. This limits conclusions in terms of the effect of residual C peptide may have on clinical outcomes.

When analysing BMI, there were only 8 recipients with T2DM *and* a BMI > 30kg/m^2^, so we advise caution when interpreting these results. It is important to consider that many of the patients in this study are on dialysis and potentially could have large fluctuations in pre-operative weight, although in general we assume a dry weight is documented and recorded. Recipient weight was taken once at the time of listing, rather than serial measurements and so may be influenced by their dialysis schedule.

A final limitation is the missing data present in post-operative complications, a common problem when utilising large databases. A complete-case analysis was chosen as our statistical method to deal with this.

This is one of the largest studies ever performed and the only study from a UK population. It supports the findings of other national, and international studies. Our study is unique as common complications after SPK transplant were also analysed, as well as the impact of BMI and ethnicity delineated by type of diabetes.

In summary, we have found no statistically significant differences in death censored pancreas graft survival, kidney graft survival or patient survival when delineating by diabetes type which is consistent with previous studies research. Despite this evidence it should be noted SPK is rarely performed for T2DM patients, more so in the UK than several other countries. We have shown that fit patients with T2DM who are insulin dependent, not overtly obese (BMI < 30 kgm^2^ although this would subject to opinion) and who are uraemic will do well with SPK. We were unable to draw useful conclusion regarding C peptide status in terms of clinical outcome.

We believe there needs to be a clear consensus on listing criteria and the diagnosis of T2DM to ensure that eligible patients are being referred for SPK transplant and are not excluded by questionable listing criteria. We also believe further research is needed within the UK population to better understand the disparity in percentage of T2DM patients receiving a SPK transplant.

## Data Availability

The data analyzed in this study is subject to the following licenses/restrictions: Access to the Dataset must be sought from the NHS Blood and Transplant UK registry team. Requests to access these datasets should be directed to statistical.enquiries@nhsbt.nhs.uk.

## Abbreviations

BMI, Body Mass Index; BAME, Black, Asian and Minority Ethnic; CIT, Cold Ischaemia Time; CVA, Cerebrovascular Accident; DBD, Donor Brain Death; DCD, Donor Circulatory Death; IPTR, International Pancreas Transplant Registry; ITA, Islet Transplant Alone; KTA, Kidney Transplant Alone; MI, Myocardial infarction; NHSBT, National Health Service Blood and Transplant; NICE, National Institute of Health and Care Excellence; PAK, Pancreas after Kidney; PTA, Pancreas Transplant Alone; PTDM, Post Transplantation Diabetes Mellitus; SIK, Simultaneous Islet and Kidney Transplant; SPK, Simultaneous Pancreas and Kidney; T1DM, Type one diabetes mellitus; T2DM, Type two diabetes mellitus; UTI, Urinary tract infection; UK, United Kingdom; UNOS, United Network for Organ Sharing; WIT, Warm Ischaemia Time.
